# Identification of genetic loci associated with major agronomic traits of wheat (*Triticum aestivum* L.) based on genome-wide association analysis

**DOI:** 10.1186/s12870-021-03180-6

**Published:** 2021-09-13

**Authors:** Woo Joo Jung, Yong Jin Lee, Chon-Sik Kang, Yong Weon Seo

**Affiliations:** 1 Department of Plant Biotechnology, Korea University, Seoul, 02841 South Korea; 2 Department of Biotechnology, Korea University, Seoul, 02841 South Korea; 3 National Institute of Crop Science, Rural Development Administration, Wanju, 55365 Republic of Korea

**Keywords:** Bread wheat, Agronomic trait, Winter survival, GWAS

## Abstract

**Background:**

Bread wheat (*Triticum aestivum* L.) is one of the most widely consumed cereal crops, but its complex genome makes it difficult to investigate the genetic effect on important agronomic traits. Genome-wide association (GWA) analysis is a useful method to identify genetic loci controlling complex phenotypic traits. With the RNA-sequencing based gene expression analysis, putative candidate genes governing important agronomic trait can be suggested and also molecular markers can be developed.

**Results:**

We observed major quantitative agronomic traits of wheat; the winter survival rate (WSR), days to heading (DTH), days to maturity (DTM), stem length (SL), spike length (SPL), awn length (AL), liter weight (LW), thousand kernel weight (TKW), and the number of seeds per spike (SPS), of 287 wheat accessions from diverse country origins. A significant correlation was observed between the observed traits, and the wheat genotypes were divided into three subpopulations according to the population structure analysis. The best linear unbiased prediction (BLUP) values of the genotypic effect for each trait under different environments were predicted, and these were used for GWA analysis based on a mixed linear model (MLM). A total of 254 highly significant marker-trait associations (MTAs) were identified, and 28 candidate genes closely located to the significant markers were predicted by searching the wheat reference genome and RNAseq data. Further, it was shown that the phenotypic traits were significantly affected by the accumulation of favorable or unfavorable alleles.

**Conclusions:**

From this study, newly identified MTA and putative agronomically useful genes will help to study molecular mechanism of each phenotypic trait. Further, the agronomically favorable alleles found in this study can be used to develop wheats with superior agronomic traits.

**Supplementary Information:**

The online version contains supplementary material available at 10.1186/s12870-021-03180-6.

## Background

Bread wheat (*Triticum aestivum* L.) is one of the most widely consumed cereal crops worldwide, providing approximately 30% of dietary energy to humans [1]. Since the first cultivation of wheat about 10,000 years ago, it has spread over all continents, a fact which has primarily been attributed to its wide adaptability to diverse environments [2]. Furthermore, the development of plant breeding contributed to the success of wheat after the Mendelian genetic law was confirmed in 1900. In particular, the “Green Revolution” between 1965 and 1985 drastically increased the wheat yield by introducing high-yielding semi-dwarf wheat varieties [3]. The yield of winter wheat in the US in 2020 was 3.42 t/ha, which is almost twice that from 50 years ago [4].

While conventional breeding is dependent on selecting superior varieties by phenotyping, more recently, with the development of sequencing technology, molecular breeding began to utilize genetic diversity among varieties. A number of molecular markers such as simple sequence repeat (SSR), random amplified polymorphic DNA (RAPD), and restriction fragment length polymorphism (RFLP) have been utilized in plant breeding [5–7]. However, since the cost of next-generation sequencing (NGS) has begun to decrease, single nucleotide polymorphisms (SNPs) are now becoming the most frequently used marker, owing to their abundance throughout all plant species [8].

Genome-wide association selection (GWAS) is a useful method for identifying candidate genes explaining phenotypic traits by testing the association between the marker type and the phenotype of individuals in a population [9]. As a genotyping tool for GWAS, genotyping-by-sequencing (GBS) is one of the popular methods, and array-based and NGS-based platforms for genome-wide high-throughput genotyping have been developed for a number of crops, such as maize [10], rice [11], and barley [12]. For wheat, several SNP genotyping arrays have been developed, including the 9 K [13], 15 K [14], 35 K [15], and 90 K [16] arrays. Among them, the 90 K iSelect SNP genotyping array, which was developed using 19 bread wheats and 18 durum wheats, is one of the most reliable and widely used tools, and a number of SNPs related to important agronomic traits, such as kernel size [17], grain yield [18], and spike related traits [19], have been identified using the array. Furthermore, with the recent announcement of the wheat reference genome [20], the functional annotation of the genes located near each SNP has become available.

In this study, we observed nine phenotypic traits of 287 wheat accessions from diverse origins in different environments. The phenotypic data were statistically analyzed, and the relationship between each trait was elucidated. Genotyping of the wheat accessions was performed using the wheat 90 K iSelect SNP genotyping array, and GWA was analyzed. From the GWA analysis, a number of significant genetic loci related to phenotypic traits and candidate genes were suggested, which will be helpful in developing agronomic traits-fortified wheats.

## Results

### Phenotypic data and correlation analysis

**Fig. 1:**
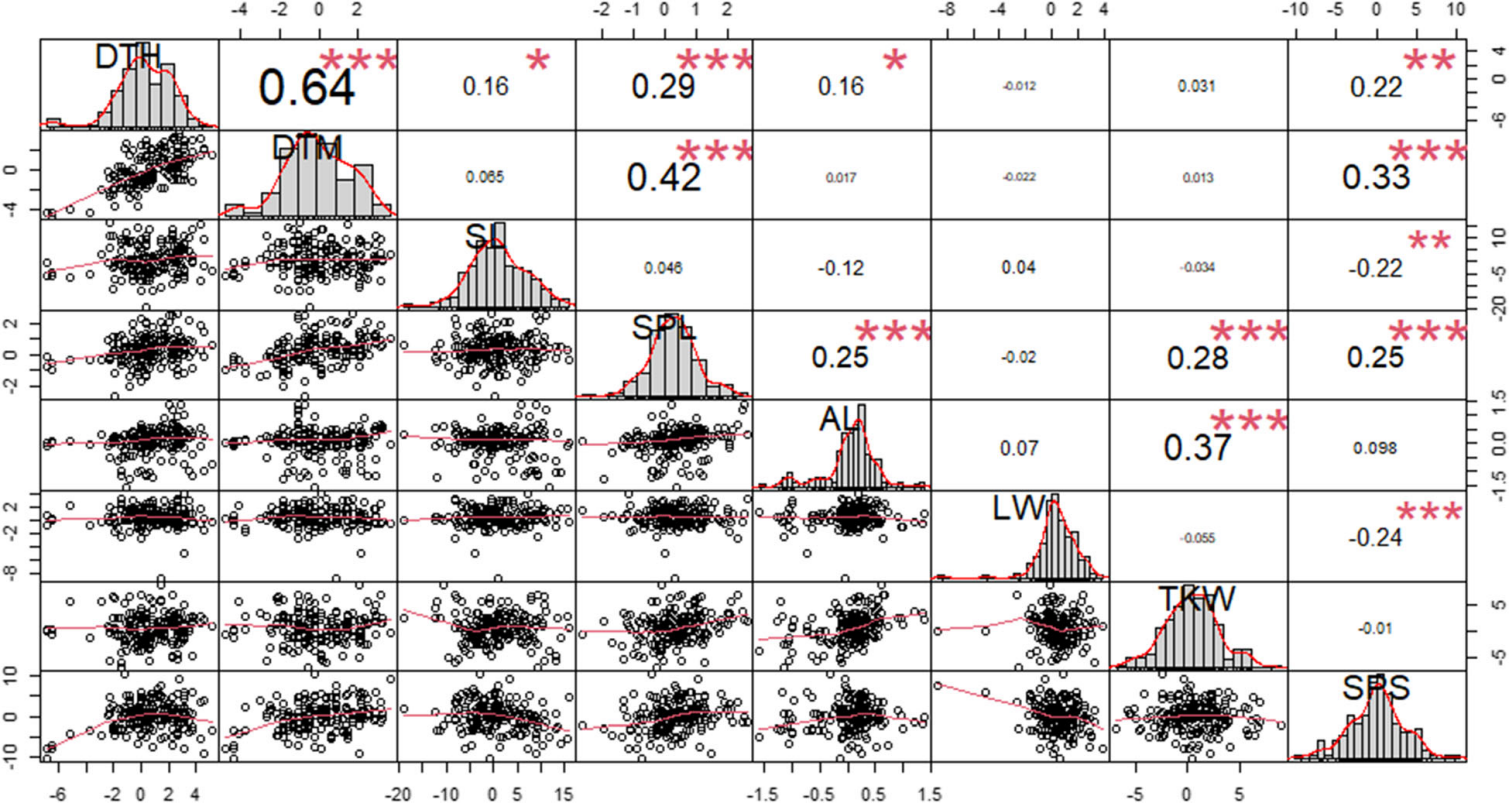
Pearson’s correlation analysis of the observed agronomic traits’ best-linear unbiased prediction (BLUP) values. The distribution of each dataset is shown on the diagonal, and the bivariate scatter plots with a fitted line and the value of the correlation (*R*^2^) plus the significance level are represented as stars on the bottom and top of the diagonal, respectively. DTH; days to heading, DTM; days to maturity, SL; stem length, SPL; spike length, AL; awn length, LW; liter weight, TKW; thousand kernel weight, SPS; the number of seeds per spike (***: *p* < 0.001, **: *p* < 0.01, *: *p* < 0.05)

A total of nine agronomic traits of 287 wheat lines were observed in two to four different environments and analyzed (Additional file 2: Fig. S1 and Table 1). It was observed that WSR had the largest variation while DTH and DTM had the smallest variation among the genotypes based on the CV. *H*^2^ ranged between 0.051 and 0.848 in which LW and DTH had the smallest and largest values, respectively. From the ANOVA analysis (Additional file 3: Table S2), it was revealed that significant differences were present among the genotypes (*p* < 0.0001) and the environments affected these traits (Genotype * Environment *p* < 0.0001). The Pearson’s correlation coefficient between each observed trait’s BLUP, except for WSR, was calculated (Fig. 1). DTH was positively correlated with DTM, SL, SPL, AL, and SPS, and DTM was positively correlated with SPL and SPS. A positive correlation was also observed between SPL and AL, TKW, and SPS, and between AL and TKW, while a negative correlation was observed between SL and SPS, and between LW and SPS.

**Table 1:** Basic statistics of the phenotypic variations observed for nine quantitative traits

Trait	Environment^a^	Genotype	Mean	SD	Min.	Max.	Median	CV	*H*^2^
Winter survival rate (0: 0–10% to 9: 90–100%)	E1	287	4.171	2.796	0	9	4.500	0.670	0.606
	E2	287	4.681	2.370	0	9	5.000	0.506	
Days to heading	E1	260	214.8	2.904	204.0	218.0	216.0	0.014	0.848
	E3	188	189.3	2.136	181.0	195.0	189.0	0.011	
	E4	188	180.1	4.056	168.0	188.0	180.0	0.023	
Days to maturity	E1	135	253.0	1.789	249.0	258.0	253.0	0.007	0.815
	E3	188	224.4	2.292	219.0	230.0	224.0	0.010	
	E4	188	217	2.904	209	222	217	0.013	
Stem length (cm)	E1	260	77.96	15.288	40.00	122.70	74.55	0.196	0.632
	E2	249	82.540	10.432	50.700	107.300	82.700	0.126	
	E3	188	77.7	9.763	51.0	106.0	77.0	0.126	
	E4	188	88.09	12.085	56.00	123.00	86.50	0.137	
Spike length (cm)	E1	259	9.449	1.723	6.000	14.300	9.500	0.182	0.773
	E2	249	9.116	1.430	3.300	13.000	9.000	0.157	
	E3	188	8.974	1.023	5.900	12.000	9.000	0.114	
	E4	188	8.719	1.060	5.500	11.800	8.700	0.122	
Awn length (cm)	E1	258	5.718	1.806	0.000	9.700	5.900	0.316	0.375
	E2	249	5.286	1.550	0.000	9.800	5.500	0.293	
	E3	188	4.915	1.675	0.000	8.300	5.100	0.341	
	E4	182	4.729	1.479	0.500	9.100	4.800	0.313	
Liter weight (g)	E1	255	825.600	53.909	460.000	960.000	828.000	0.065	0.051
	E3	188	789.7	19.648	731.0	833.0	793.0	0.025	
Thousand kernel weight (g)	E1	269	37.830	5.952	24.000	54.000	37.800	0.157	0.641
	E3	188	41.65	4.180	30.90	53.30	41.70	0.100	
Number of seeds per spike	E1	169	52.770	13.283	26.300	91.300	52.300	0.252	0.421
	E3	188	35.27	5.731	15.00	51.00	36.00	0.163	

^a^ E1: Deokso in 2018–2019 season, E2: Deokso in 2019–2020 season, E3: Jeonju in 2018–2019 season, E4: Jinju in 2018–2019 season

### Genome-wide distribution of SNP markers

Of the 81,587 SNP markers from the wheat 90 K iSelect array used in the genotyping, 30,218 remained after removing the markers with minor allele frequency < 0.05 and missing data > 10% (Additional file 4: Table S3). The SNPs were distributed on all the chromosomes, with 5496; 5853; 3984; 2565; 4313; 3935; and 3900 on chromosomes 1–7, respectively. A total of 172 SNPs were not physically mapped onto the wheat reference sequence. When comparing the subgenomes, the markers were mostly positioned on the A and B genomes, with 13,408 and 12,629, respectively, whereas only 4009 markers were located on the D genome. The range of the locations of the markers on the chromosomes was diverse, with the smallest for 1D from 31.5 to 498,397 kb and the largest for 3B from 240.2 to 851,642 kb.

### Population structure and linkage disequilibrium (LD)

**Fig. 2:**
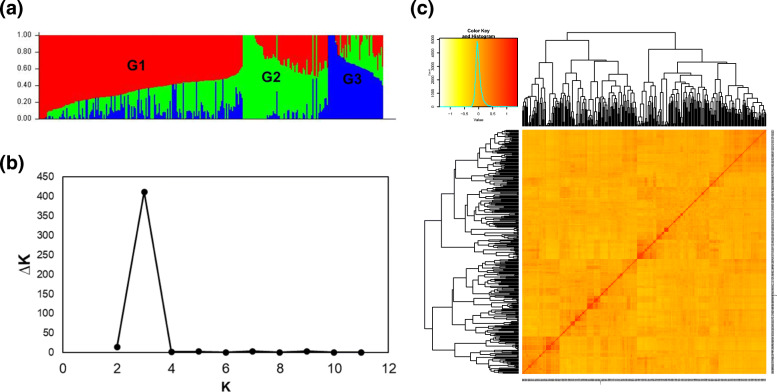
Population structure and diversity analysis of 287 wheat genotypes used in this study based on 30,217 SNP markers. **a** Population structure based on the STRUCTURE program when *K* = 3. G1, G2 and G3 represent each sub-population. **b** Δk over three repeats of structure analysis. **c** Kinship matrix of 287 wheat genotypes based on the TASSEL5 program

The population structure of the 287 wheat genotypes was investigated following the ΔK method, followed by validation via PCA and neighbor-joining kinship matrix analysis. The outcomes of the three analyses indicated that the genotypes could be divided into three subgroups based on their genotypes (Fig. 2 and Additional file 1: Table S1). The largest group (G1) contained 168 genotypes, and G2 and G3 consisted of 72 and 46 genotypes, respectively. The wheat genotypes derived from the same country of origin were usually in the same subgroup. The wheats originating from Australia, Austria, Bulgaria, Canada, Ethiopia, Hungary, India, Ukraine, and Uzbekistan belonged to G1, while those from Afghanistan, Argentina, Colombia, and Russia belonged to G2. Some of the wheats that came from China, Japan, Mexico, North Korea, and the USA were in both G1 and G2. The Korean breeding lines were divided into three subgroups, but those with “Keumgang” wheat as an ancestor mostly belonged to G3.

From the LD decay analysis, critical r^2^ value 0.34 was identified for all wheat genotypes by taking the 95th percentile of the coefficient square (represented by the red dashed line in Additional file 9: Fig. S4). The highest number of pair-markers were found on the A genome (44%) followed by the B genome (42%) and the D genome (14%). It was observed that LD decay distance in D chromosome was longer than that of A and B chromosome as shown in Fig. S4. The LD decay was constructed using chromosomal distance and the critical r^2^ value as the threshold to indicate the LD decay length, which attained 250 kb for the whole genome.

### Genome-wide association and gene expression analysis

**Fig. 3:**
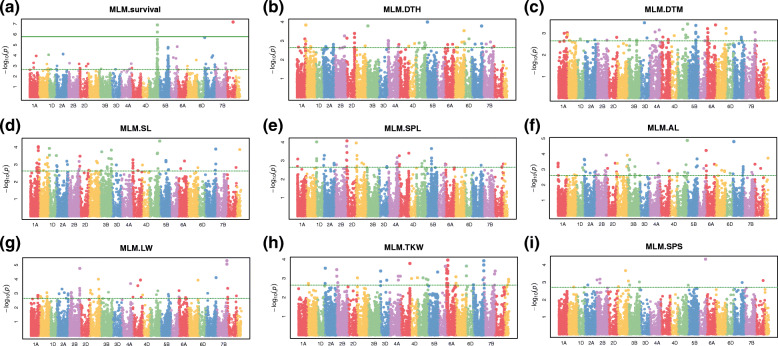
Summary of genome-wide association plots for statistically significant *P* values across 21 wheat chromosomes for SNP markers associated with nine agronomic traits. **a** winter survival rate, **b** days to heading, **c** days to maturity, **d** stem length, **e** spike length, **f** awn length, **g** liter weight, **h** thousand-kernel weight, **i** the number of seeds per spike. X- and Y-axes represent the SNP markers along each wheat chromosome and the -log_10_(*P*-value), respectively. The dotted lines designate -log_10_1E^− 04^ threshold for significant associations

The MTAs of nine agronomic traits were identified via MLM with K + Q or K + P, followed by inspection of Q-Q plots and Manhattan plots. The MTAs with -log_10_P > 3 were designated as significant, and the MTAs identified in both the K + Q and the K + P analysis were identical. The Manhattan plots of MTAs were revealed from the K + Q analysis, and the Q–Q plot and MAF plot of the SNPs are shown in Fig. 3 and Additional file 5: Fig. S2, respectively. The number of significant MTAs identified in each agronomic trait were 63, 20, 17, 31, 23, 40, 24, 31, and 6 for WSR, DTH, DTM, SL, SPL, AL, LW, TKW, and SPS, respectively (Additional file 7: Table S4). The chromosomal distribution of the MTAs in each trait was diverse among the chromosomes, as shown in Additional file 7: Table S4.

The genes located within 250 kb of each significant locus were searched and annotated using the IWGSC Wheat RefSeq v1.0. Based on the public RNAseq data, a total of 28 genes with more than 2-fold gene expression increase under cold treatment or with the relevant tissue-specific expression were selected (Table 2 and Additional file 6: Fig. S3). For WSR, ten genes closely located to the significant MTAs were shown to respond to cold temperatures. Most of the genes were found to be located on chromosome 5, and each one was located on chromosome 2 and 4, respectively, while two genes were located on chromosome 7. For DTH, three genes specifically expressed in the stem axis, flag leaf, or peduncle were selected and located on chromosomes 2, 6, and 7. For DTM, nine genes highly expressed in the grain or spike were screened, among which three were located on chromosome 4, while one and five genes were located on chromosomes 5 and 6, respectively. For SL, two genes representing specific expression in the internode and peduncle were discovered, each of which was located on chromosomes 4 and 5, respectively. For SPL, one gene was found to be highly expressed in spike, which was localized on chromosome 2. For AL, one F-box family protein encoding gene with high expression in spikelets and awn on chromosome 2 was observed. For SPS, two genes specifically expressed in grains were discovered, located on chromosomes 2 and 3.

**Table 2:** The significantly agronomic traits-associated markers and the putative genes concerned with the agronomic traits

Gene ID	Annotation	Associated SNP	SNP position on chromosome	***P***-value	Alleles	MAF
**Winter survival rate**						
TraesCS2D01G032800	GroES-like zinc-binding alcohol dehydrogenase family protein	RAC875_c38003_164	2D:13007354	0.0008	G/A	0.21724
TraesCS4A01G469900	Homeobox BEL1-like protein	Kukri_c18677_237	4A:731352373	0.0008	A/G	0.36738
TraesCS5A01G359900	Phosphatase 2C family protein	RAC875_c23340_2243	5A:561565292	0.0008	A/G	0.3723
TraesCS5A01G360000	Low temperature and salt responsive protein	RAC875_c23340_2243	5A:561565292	0.0008	A/G	0.3723
TraesCS5A01G391700	Vrn-A1	IAAV3043	5A:586537895	0.0003	A/C	0.08451
TraesCS5A01G394900	UDP-glucose 6-dehydrogenase	BobWhite_c471_2245	5A:588454245	0.0002	A/G	0.44624
TraesCS5B01G397500	ABC transporter B family protein	BS00065936_51	5B:574848793	0.0002	A/G	0.16135
TraesCS5B01G454100	Protein kinase family protein	wsnp_Ku_rep_c102339_89347150	5B:626668012	0.0003	A/G	0.12121
		wsnp_Ex_c53426_56666788	5B:626666488	0.0007	G/A	0.15548
TraesCS7A01G389900	Extracellular ligand-gated ion channel protein	BS00024617_51	7A:566002702	0.0001	A/G	0.21715
TraesCS7D01G357200	Transmembrane protein, putative	Ra_c51831_507	7D:460532222	6.71E-08	G/A	0.0669
**Days to heading**						
TraesCS2D01G468900	Starch synthase family protein	CAP11_c1070_54	2D:577071973	0.0002	G/A	0.42179
TraesCS6D01G212200	Phenylalanine ammonia-lyase	RAC875_c26887_562	6D:300325843	0.0009	G/A	0.09666
TraesCS7A01G484300	Receptor-like kinase	wsnp_Ku_c42539_50247597	7A:675380487	0.0002	C/A	0.08772
**Days to maturity**						
TraesCS4B01G129300	Exostosin family protein	RAC875_c35152_372	4B:168404351	0.0009	A/G	0.11228
TraesCS4B01G225800	Proline-rich protein	Kukri_c5502_2513	4B:534190263	0.0009	A/C	0.31317
TraesCS4B01G226000	Proline-rich protein	Kukri_c5502_2513	4B:534190263	0.0009	A/C	0.31317
TraesCS5B01G356300	UTP--glucose-1-phosphate uridylyltransferase	RAC875_c30584_75	5B:536161912	0.0004	C/A	0.41472
TraesCS6A01G117600	Homeobox protein	wsnp_BG262421A_Ta_2_2	6A:87936718	0.0009	G/A	0.15114
TraesCS6A01G406900	F-box family protein	RAC875_c14887_829	6A:612287507	0.0002	G/A	0.19965
TraesCS6A01G407000	F-box family protein	RAC875_c14887_829	6A:612287507	0.0002	G/A	0.19965
TraesCS6A01G407500	F-box family protein	RAC875_c14887_829	6A:612287507	0.0002	G/A	0.19965
TraesCS6A01G407600	F-box family protein	RAC875_c14887_829	6A:612287507	0.0002	G/A	0.19965
**Stem length**						
TraesCS4B01G052400	MYB transcription factor	Excalibur_c36630_2194	4B:41020506	0.0007	G/A	0.20285
TraesCS5A01G320500	Myb factor	Tdurum_contig30483_167	5A:533701309	0.0004	A/G	0.49734
**Spike length**						
TraesCS2D01G036800	Cullin-associated NEDD8-dissociated protein 1	BS00062567_51	2D:13994909	0.0008	A/G	0.14312
**Awn length**						
TraesCS2D01G064400	F-box family protein	Tdurum_contig42153_6232	2D:27609458	0.0009	G/A	0.08673
**Number of seeds per spike**						
TraesCS2B01G087000	Response regulator 1	BS00067781_51	2B:48944396	0.0006	C/A	0.21617
TraesCS3A01G311000	Cytokinin oxidase/dehydrogenase	RFL_Contig2616_1422	3A:549908041	0.0001	G/A	0.05944

Real-time PCR was performed to validate the gene expression of the candidate genes. At least two genes for each trait were randomly selected and gene expression was observed. For WSR, *TraesCS5A01G391700*, *TraesCS5A01G394900*, and *TraesCS5B01G454100* showed increased gene expression at 24 h, 12 h, and 12 h after cold treatment (Additional file 10: Fig. S5). For DTH, *TraesCS6D01G212200* and *TraesCS7A01G484300* represented high expression in stem and peduncle, or only in peduncle, respectively (Fig. S6A). For DTM, *TraesCS4B01G225800* and *TraesCS6A01G407500* were highly expressed in spike (Fig. S6B). For SL, *TraesCS4B01G052400* represented high expression in peduncle, and *TraesCS5A01G320500* showed high expression in stem followed by peduncle and spike (Fig. S6c). For SPL and AL, *TraesCS2D01G036800* and *TraesCS2D01G064400* were highly expressed in spike and flag leaf, respectively (Fig. S6d&e). For SPS, *TraesCS2B01G087000* represented the highest expression in spike, and *TraesCS01G311000* was also highly expressed in spike (Fig. S6f).

### The effect of alleles on agronomic traits

**Fig. 4:**
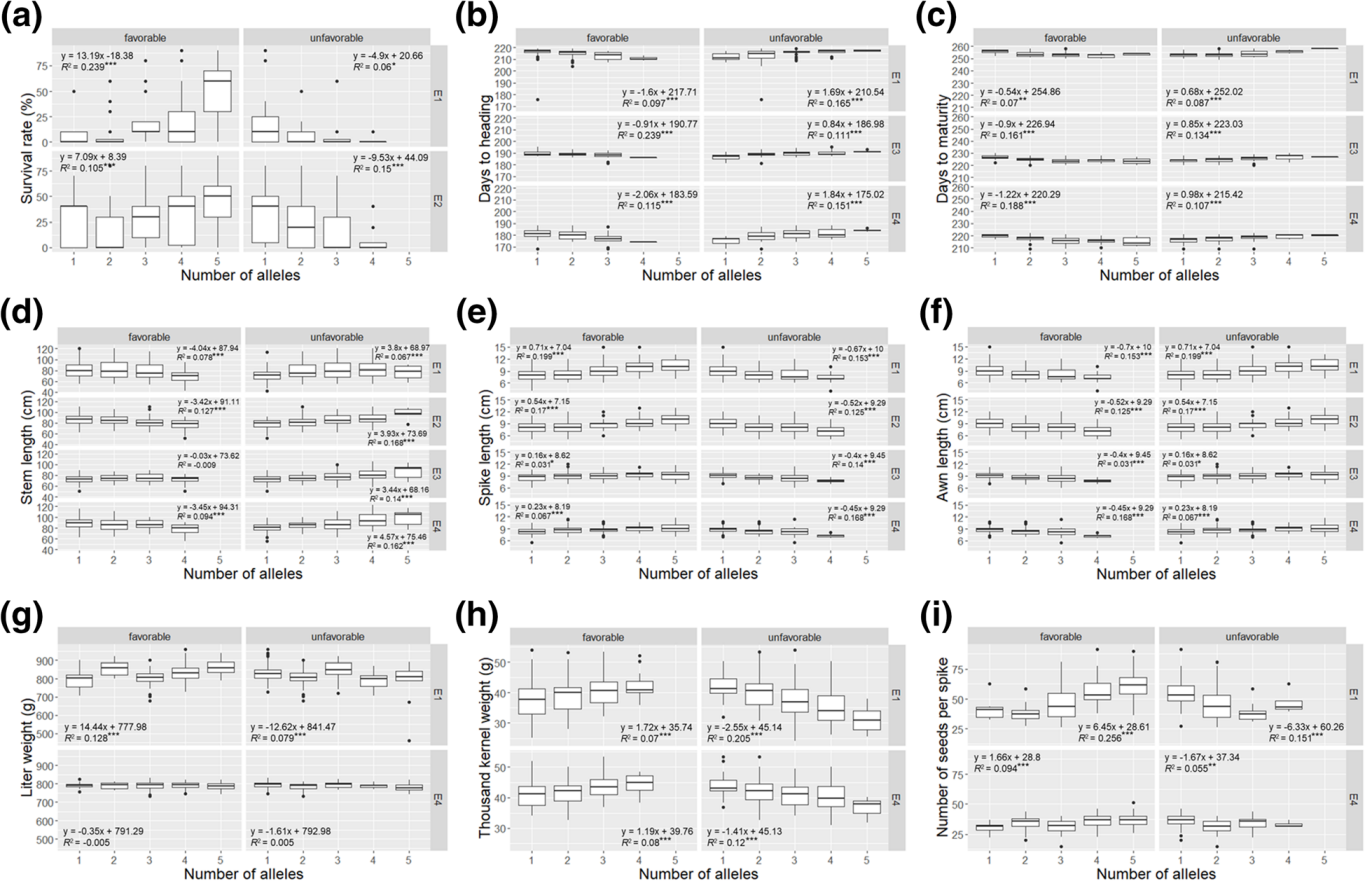
The effect of alleles on the agronomic traits in different environments. The numbers on the X-axes indicate the number of the favorable or unfavorable alleles, while the Y-axes represents the phenotypic data distribution of the genotypes possessing each number of alleles. The bottom, middle and top lines in each boxplot represent 25, 50 and 75% of the phenotypic data, respectively. E1: Deokso in 2018–2019 season, E2: Deokso in 2019–2020 season, E3: Jeonju in 2018–2019 season, E4: Jinju in 2018–2019 season. The equation of regression line between each phenotypic data and the number of alleles, and the adjusted R-square values are represented (***: *p* < 0.001, **: *p* < 0.01, *: *p* < 0.05)

To investigate the allele effect on the observed agronomic traits, the phenotypic data distribution of the wheat genotypes possessing different numbers of favorable or unfavorable alleles in different environments were compared (Fig. 4). In this study, the high winter survival rate, shorter days to heading and maturity, short stem length, spike length, short awn length, heavy liter weight, and thousand kernel weight, and large number of seeds per spike were considered favorable. In most cases, the number of favorable/unfavorable alleles had a significant positive or negative effect on each agronomic trait, except for the unfavorable alleles for WSR, SPL, and SPS, and the favorable alleles for DTH, SL, AL, and TKW, for which there were no wheat genotypes possessing all five unfavorable or favorable alleles. Furthermore, the allele effect for each agronomic trait was observed regardless of different environments, except for the favorable alleles in E3 for SL and the favorable/unfavorable alleles in E4 for LW.

## Discussion

In this study, nine phenotypic traits of wheat were observed in two to four different environments and utilized in the GWA analysis (Additional file 2: Fig. S1). WSR was observed in four environments; however, in E3 and E4, there was little difference among the genotypes due to their warm climate condition, and these were therefore removed from the analysis. DTH and DTM represented large variations depending on the different environments. It was shown that they had higher values in the cold environment (E1) than in the warm environment (E4). In the Pearson’s correlation analysis between each phenotypic trait (Fig. 1), DTH and DTM were highly correlated with each other, and also represented a positive correlation with SPL and SPS. This is consistent with previous studies [21, 22], which might indicate that nutrient absorption during the longer vegetative development period results in a larger spike length, and eventually a larger number of seeds per spike. A positive correlation between AL and TKW was also observed in a previous study [23], which still needs to be further studied.

The relationships among the genotypes were analyzed by three different methods: structure subpopulation analysis, PCA, and kinship matrix (Fig. 2 and Additional file 1: Table S1). All three analyses produced the same three subpopulations, which proves the reliability of the genotype analysis. It was thus shown that the genotypes were mostly divided into two subpopulations G1 and G2, while G3 was mostly composed of KU-developed lines possessing “Keumgang” as a maternal line. Since the phenotypic traits of the wheats in G3 were diverse, the focused investigation of these lines would help to identify a major QTL in a similar genetic background.

The LD analysis showed that the paired markers were relatively less in D chromosomes and LD decay distance was also especially longer in D chromosomes (Fig. S4). This phenomenon was observed in a previous study [21]. This might be due to small number of SNPs in D chromosomes in the iSelect 90 K chip, which had been caused by little polymorphism in D genome among wheat genotypes [16]. This is thought to be natural since D genome had been relatively recently incorporated into wheat genome after the mix of A and B genome [1] so that D genome had little chance to diverge than A and B genome. The LD decay distance was estimated to be 250 kb, which was higher than 74.7 kb of [18] and lower than 393 kb of [24].

GWA analysis and candidate gene search for MTA identified a number of genes with functions relevant to phenotypic traits (Table 2 and Additional file 6: Fig. S3). Ten genes were identified for WSR. Six out of ten genes were located on the long arm of chromosome 5 possessing *Fr-1* and *Fr-2* locus, which controls cold tolerance [25]. An earlier GWAS study regarding cold tolerance also reported that significant MTAs were mostly located on the long arm of chromosome 5 [24]. They found polymorphisms in *CBF* genes, an important transcription factor regulating cold response pathway [25], but MTAs regarding *CBF* genes were not identified in this study. Instead, other putative candidate genes in chromosome 5 were suggested according to previous studies. *TraesCS5A01G359900* (Phosphatase 2C family protein) belongs to the major group of protein phosphatases in plants and plays multiple roles in diverse plant metabolism. It was found that rice PP2C negatively regulates ABA signaling and enhances abiotic stress tolerance in *Arabidopsis* [26]. *TraesCS5A01G360000* (low temperature and salt responsive protein) was extremely highly expressed under cold temperature, and the protein is conserved in diverse species; however, its function still needs to be elucidated [27]. *TraesCS5A01G391700* (*Vrn-A1*) controls flowering time and is located in *the frost Resistance-1* (*Fr-1*) locus, which is reported as a major QTL for cold tolerance [28], and is now thought to have pleiotropic effects in both traits [29]. *TraesCS5A01G394900* (UDP-glucose 6-dehydrogenase) is involved in sucrose/polysaccharide metabolism and cell wall biosynthesis, and the overexpression of UDP-glucose dehydrogenase from *Larix gmelinii* in *Arabidopsis* enhanced cold tolerance [30]. *TraesCS5B01G397500* (ABC transporter B family protein) is a large ABC transporter family protein, which functions as a plasma membrane modifier that could activate the biotic and abiotic stress responses [31]. *TraesCS5B01G454100* (protein kinase family protein) is involved in multiple plant metabolism pathways, and several protein kinases such as calcium-dependent protein kinase (CDPK), CBL-interacting protein kinase (CIPK), and mitogen-activated protein kinase (MAPK) have been identified to regulate cold tolerance in diverse plant species [32]. Putative candidate genes on other chromosomes were also suggested. *TraesCS2D01G032800* (GroES-like zinc-binding alcohol dehydrogenase family protein) is an alcohol dehydrogenase family in wheat, and it was previously predicted that this gene family may play an important role in anaerobic waterlogging stress [33]. *TraesCS4A01G469900* (Homeobox BEL1-like protein) is a BEL1 transcription factor that interacts with the KNOTTED protein and responds to wound response, as studied in potato [34]. *TraesCS7A01G389900* (Extracellular ligand-gated ion channel protein) is not well characterized in plants, but Ca^2+^ ion increase inside the cell is thought to be a triggering factor of the cold response pathway [35], which makes it a putative ion channel for Ca^2+^ influx. *TraesCS7D01G357200* (Transmembrane protein) is not characterized, but the cell membrane is the key organelle in cold perception [35] and several transmembrane proteins have been shown to control cold tolerance [36, 37].

For DTH and DTM, three and nine putative genes were suggested, respectively. DTH and DTM are highly quantitative traits and also significantly affected by growing environments. GWAS studies regarding DTH and DTM have been performed earlier by diverse research groups [21, 38, 39], but there were no overlapped candidate genes and a number of MTAs were identified. The putative candidate genes we suggest are known to have functions related to flowering and maturing, but overlapped genes with the previous studies were also not identified. *TraesCS2D01G468900* (Starch synthase family protein) was highly expressed in the stem axis and peduncle; this gene controls the synthesis of amylose in starch granules, and it has been suggested that it could increase sugar mobilization at floral transition, thus being involved in florigenic signaling [40]. *TraesCS6D01G212200* (Phenylalanine ammonia-lyase) is mainly involved in the lignin biosynthesis pathway, but its knock-down *Brachypodium* mutant revealed a late flowering phenotype [41], which might be related to flowering time in plants. *TraesCS7A01G484300* (receptor-like kinase) also represented peduncle-specific gene expression. Several receptor-like kinases have been found to be involved in flowering by interacting with the main flowering regulators [42], which requires further investigation of this gene. *TraesCS4B01G129300* (Exostosin family protein) was mainly expressed in the spike and anther. The function of this gene has not yet been elucidated in plants, but its expression in the pistil in tomato [43] and in the leaf at floral transition in maize [44] was observed, which implies its involvement in flowering regulation. *TraesCS4B01G225800* and *TraesCS4B01G226000* encode Proline-rich proteins that are specifically expressed in the spike. It has been suggested that the tomato hybrid proline-rich protein regulates flower abscission in tomato by controlling the ethylene response [45], which should be studied in wheat as well. *TraesCS5B01G356300* (UTP--glucose-1-phosphate uridylyltransferase) was expressed in the anther and the grain, which was proposed as a vegetative and reproductive phase rate-limiting factor in *Arabidopsis* [46]. *TraesCS6A01G117600* (Homeobox protein) was expressed in developing grains, and overexpression of the WUSCHEL homeobox transcription factor OsWOX13 in rice revealed an early flowering phenotype [47]. *TraesCS6A01G406900*, *TraesCS6A01G407000*, *TraesCS6A01G407500*, and *TraesCS6A01G407600* encode F-box family proteins that are highly expressed in the spike or grain. F-box protein is known to be involved in diverse molecular pathways, and its regulation of flowering and spike development has been observed in a number of plant species, including wheat [48].

For SL, two MYB transcription factors, *TraesCS4B01G052400* and *TraesCS5A01G320500,* were found to be highly expressed in the peduncle and internode. Plant height of wheat is known to be mainly controlled by *Reduced Height* (*Rht*) genes through regulating the pathway of hormones, reactive oxygen species, and cell wall structure [49]. Previous studies represented multiple candidate genes that could be involved in the pathway under *Rht* gene, such as auxin binding protein and protein kinase [21], zinc finger protein [39], bHLH74, GDP-mannose transporter and etc. [50] and we suggest MYB transcription factors as such candidate genes. Studies regarding MYB transcription factor in wheat have mostly concentrated on diverse abiotic stress tolerance [51]. However, it has been reported that the novel MYB-like transcription factor OsMPH1 (MYB-like gene of Plant Height 1) regulates internode cell size and eventually plant height. Further studies to investigate MYB transcription factor function in plant height regulation should be performed in the future.

Two single genes were identified as having a putative role in regulating SPL and AL, respectively. Wheat spike morphology is known to be controlled by at least three loci, Q, C, and S, which resides on chromosome 5A, 2D, and 3D, respectively [52]. These genes affect various spike traits including spike length, spike morphology, grain size and shape, but since all known common wheats have *QcS* genotype, it is likely that there are other genes which contribute to spike morphology [53]. For awn length, three dominant awn length inhibitors, *Hooded* (*Hd*), *Tipped1* (*B1*), and *Tipped2* (*B2*) are known but their functions have not been characterized yet [54]. *TraesCS2D01G036800* (Cullin-associated NEDD8-dissociated protein 1) has not been widely studied, but in tomato, the suppression of this gene resulted in several phenotypic changes, including dwarfism, early flowering, and suppression of seed germination [55]. Its function in wheat, especially governing spike development, still needs to be uncovered. *TraesCS2D01G064400* (F-box family protein) is involved in diverse plant metabolism as described above, but its role in awn length has not been reported. Nevertheless, another GWAS study in wheat suggested two MYB transcription factors as putative regulators of awn length [56], which might imply another uncharacterized role of MYB transcription factors.

For SPS, two genes showing high expression in grain were suggested as candidate genes. SPS is positively correlated with the number of spikelets per spike [57], and it is highly affected by biotic or abiotic stresses, which makes it difficult to find genetic factor determining its trait. Numerous MTAs or candidate genes were identified in previous studies, MTAs in chromosome 2A, 4A [58], 7B [19], and bHLH-encoding gene in chromosome 5D [39]. Our analysis revealed two genes in chromosome 2B and 3A, respectively. *TraesCS2B01G087000* (Response regulator 1) is one of the signal transduction response regulators with an unknown function. One of the response regulators, the phosphate starvation response regulator PHR1, is known as a regulator of the phosphate starvation response in plants, and it was revealed that in wheat the overexpression of *Ta-PHR1-A1* increased grain yield by increasing the grain number per spike [59]. *TraesCS3A01G311000* (cytokinin oxidase/dehydrogenase) regulates plant growth and development by controlling cytokinin levels in plants. Previous studies found that the gene expression levels of the cytokinin oxidase genes *TaCKX2.1* and *TaCKX2.2* were correlated with the grain number per spike [60] and *TaCKK2.4*-silenced wheat lines represented significantly increased grain numbers per spike [61].

The GWA analysis was performed using the BLUP values from the combined environments or from the separate environments (E1 to E4). The consistency of the MTAs was verified as shown in Additional file 7: Table S4. However, the markers for some traits such as DTM in E1, AL in E4, and LW in E4 were not shown in the combined environment. It might be because these traits are more variable depending on the environments than the other traits and also the number of the investigated accessions are different in each environment, so that significance of each marker can be different in other environments.

Real-time PCR was conducted to validate the stress-responsive or tissue-specific gene expression of the candidate genes *in planta* (Fig. S5 and S6). The candidate genes related to WSR were responsive to cold stress (Fig. S5), and tissue-specificity of the other genes’ expressions were mostly consistent with those of the RNAseq data (Fig. S3). Especially, *TraesCS2B01G087000* represented dramatically high expression in spikes, approximate 140,000 times higher than in leaves, which makes it a promise candidate gene for SPS (Fig. S6f). However, *TraesCS2D01G064400* expression did not show a great difference among the tissues, which could be because awn tissue was not examined in the real-time PCR analysis (Fig. S6e). Further experiments will be needed to characterize the function of *TraesCS2D01G064400*.

The allele effect of the MTAs in each agronomic trait was observed (Fig. 4). The accumulation of the favorable alleles was positively associated with a higher WSR, shorter DTH, DTM, and SL, longer SPL and AL, higher LW, TKW, and SPS, and the accumulation of unfavorable alleles negatively affected those traits. The allele effect was observed in most of the environments, except for in E3 for SL and in E4 for LW. This might be partially due to low *H*^2^ in LW (Table 1), which implies that these traits are highly affected by environments rather than that by genotypes.

## Conclusions

In the present study, GWA analysis was performed for nine phenotypic traits of wheat in the field, and a total of 254 significant MTAs and 28 candidate genes were predicted for the seven traits, including winter survival rate, days to maturity, stem length, spike length, awn length, liter weight, and seeds per spike. Previously identified genes with known functions were observed, and several novel genes with possible uncharacterized functions in each trait were also identified. Further studies of these candidate genes and the utilization of the significant SNP markers will help to verify their molecular functions in the relevant trait and also to develop agronomic traits-improved wheats.

## Methods

### Plant materials and phenotype measurements

In this study, 287 wheat cultivars and advanced lines were planted at the Korea University Experimental Field Station in the Deokso area (N37.35°, E127.14°, Elevation = 62 m) in the 2018–2019 (E1) and 2019–2020 (E2) growing seasons. A total of 189 lines were also planted in the test field of the National Institute of Crop Science of Korea in Jeonju (E3; N35.50°, E127.02°, Elevation = 32 m) and Gyeongsangnam-do Agricultural Research & Extension Services in Jinju (E4; N35.12°, E128.06°, Elevation = 20 m) during the 2018–2019 growing season. The field experiments were in accordance with local legislation of Korea government. Wheat accessions indicated as “Developed in KU” in “Country of origin” column are breeding lines developed in Korea University. Any accessions indicated as numbers in “IT number” column are germplasms that were kindly provided by National Agrobiodiversity Center in Rural Development Administration, Korea (Additional file 1: Table S1). The materials were planted on the 4th, 25th, and 29th of October in 2018, and harvested in June 2019, when the plants were fully matured in Deokso, Jeonju, and Jinju, respectively. For the 2019–2020 growing season in Deokso, the wheat was planted on the 17th of October, 2019 and harvested in June 2020. Five grams of seeds from each germplasm were planted in a 1.2 m row spaced 40 cm apart, and the experiment was conducted in two replications following alpha lattice design in each region.

Phenotype measurements were performed as follows. Freezing tolerance was evaluated as winter survival in integers representing the survival rate of plants per genotype, ranging from 0 (0–10%) to 9 (90–100%) after winter. Heading and spike maturity dates were recorded when spikes of half of each germplasm had emerged or browned, respectively. DTH and DTM were calculated by subtracting the planting date from those dates. Three plants were randomly selected and SL was measured from the ground to the bottom end of the spike. The main spikes of three plants were randomly selected and the SPL was measured from the base of the rachis to the topmost spikelet, and AL was measured. SPS was counted for three spikes in each germplasm. For LW, 200 mL of seed weight of each line was measured and multiplied by five. For TKW, 200 seeds of each line were counted and the weight was multiplied by five.

### Phenotypic data analysis

All phenotypic data analyses were conducted using R version 4.4.0 (R Core Team). The mean, standard deviation, median, and coefficient of variation (CV) of quantitative data in each environment were calculated. For each accession, BLUP (best linear unbiased prediction) values across all environments were calculated using the “lme4” package [62] in R and the broad sense heritability (*H*^2^) was estimated on the basis of entry mean following V_G_/[V_G_ + (V_E_/y) + V_error_], where V_G_ is the genotypic variance, V_E_ is the environment variance. V_error_ is the residual error variance, and y is the number of environments. Analysis of variance (ANOVA) was conducted by including the genotypes, environment, and genotype × environment interactions as random factors. Pearson pairwise correlation was calculated for all BLUP traits using the “cor” function and the “PerformanceAnalytics” package [63] in R.

### Genotyping and SNP calling

For the genotyping assay, leaves were sampled from the wheats before the booting stage in the Deokso field and stored at − 80 °C until use. DNA was extracted from a single plant of each germplasm, according to the USDA instructor’s manual using the CTAB method [64]. DNA was sent to the USDA-ARS Small Grain Genotyping Center, Fargo (https://wheat.pw.usda.gov/GenotypingLabs/fargo) for use in the Illumina iSelect 90 K SNP Assay. SNP allele clustering and genotype calling were performed with the GenomeStudio Module Polyploid Genotyping 2.0 software (https://support.illumina.com/downloads/genomestudio-2-0.html). Markers with minor allele frequencies < 0.05 and missing data > 10% were removed, giving a total of 30,218 high-quality SNPs for use in the population structure analyses and genome-wide association (GWA) analysis.

### Population structure and linkage disequilibrium

The model-based Bayesian cluster analysis program STRUCTURE v2.3.4 [65] was used to infer the population structure. A total of 10,000 burn-in periods followed by 100,000 Markov chain Monte Carlo (MCMC) iterations from K = 2 to K = 12 clusters were used to identify the optimal cluster (K). Three independent runs were generated for each K. An ad hoc quantity statistic, ΔK, based on the rate of change in the log probability of data between successive K values [66] was used to predict the real number of subpopulations. Principal component analyses (PCAs) were also conducted using the TASSEL v5.2.57 [67].

The linkage disequilibrium (LD) between the pairs of 30,218 SNP marker was estimated using the TASSEL v5.2.57 with a sliding window size cut off 100. The LD decay was analyzed as per physical distance according to [68]. Briefly, LD was estimated separately for unlinked loci and for loci on the same chromosome (unlinked *r*^2^ and syntenic *r*^2^, respectively). Syntenic *r*^2^ was plotted against physical distance on chromosomes and a smooth line was drawn using the “ggplot2” package [69] in R. Unlinked-*r*^2^ estimates were square root transformed and then beyond the parametric 95th percentile of that distribution was likely to be caused by genetic linkage. The intersection of the smooth line to syntenic *r*^2^ with this baseline was considered as the estimate of the extent of LD in the chromosome.

### Genome-wide association analysis

The BLUP of each accession was used to fit a mixed linear model (MLM) by applying the residual maximum likelihood (REML) algorithm, which was calculated to analyze the phenotypic data and estimate the mean of each individual over different environments. An association test was performed using the GAPIT package v3 [70] in R, and the MLM model utilized trait data with population structure and PCA to find marker-trait association. The analysis was performed twice for each trait by either K + Q (kinship and population structure) or K + P (kinship and principal component), which were compared with each other. A threshold *P*-value of 0.001 (−log_10_(P) = 3) was used to declare significant SNPs for GWAS results and the SNPs having *P*-value less than 0.001 were selected. Furthermore, the entire analysis was conducted in each different environment to validate the consistent performance of the marker-trait associations. Quantile-quantile plots were drawn based on the observed and expected log_10_(P) values.

To identify genes related to each agronomic trait, the high-confidence annotated genes located ±250 kb proximal to each identified MTA (marker-trait association) were retrieved from Ensembl Plants (http://plants.ensembl.org/index.html), and the annotation of each gene followed the International Wheat Genome Sequence Consortium (IWGSC) Wheat RefSeq v1.0. Furthermore, the gene expression of the retrieved genes was observed using public RNA-seq data. For WSR-related genes, the RNAseq of 2-week cold-treatment wheat seedlings was available at the Wheat Expression Browser (http://www.wheat-expression.com/) and those genes with a more than 2-fold increase were retrieved. For the genes concerning the rest of the agronomic traits, except for LW and TKW, tissue-specific gene expression was observed at the Wheat eFP Browser (http://bar.utoronto.ca/efp_wheat/cgi-bin/efpWeb.cgi) and the genes with TPM (Transcripts Per Million) < 5 were removed. The heatmap of gene expression was created using the Heatmapper (http://www.heatmapper.ca/).

Furthermore, to verify allele effects on each agronomic trait, the favorable and unfavorable alleles of the five most significant MTAs in each trait were retrieved. Then, the phenotypic data distribution of the lines possessing one to five of each favorable or unfavorable allele was compared in different environments. The regression analysis between the number of favorable/unfavorable alleles and each phenotypic data was conducted using R, and the boxplots were created using the “ggplot2” package in R.

### Real-time PCR

Gene expression of the candidate genes was observed in specific tissue or under cold temperature. RNA was extracted from specific tissues of wheat at heading stage, which were the first leaf from the ground, stem, peduncle, flag leaf, and spike. In order to observe the gene expression under cold temperature, three-leaf stage wheat seedlings grown at 25 °C were exposed to 4 °C. The RNA extraction was conducted using TRIzol (Thermo Fisher Scientific Co., USA) and cDNA was synthesized using the PrimeScript™ 1st strand cDNA synthesis kit (Takara Bio Inc., Japan) according to the manufacturers’ manuals, respectively. Each gene specific PCR primers was designed using the NCBI Primer-BLAST (https://www.ncbi.nlm.nih.gov/tools/primer-blast/index.cgi? LINK_LOC=BlastHome) (Table S5). Real-time PCR was performed using BrightGreen 2X qPCR MasterMix (ABM, Canada) in a CFX-96 real-time PCR machine (Bio-Rad, USA). The Ct values obtained for each gene were normalized depending on the internal gene control, and relative gene expression levels were calculated using the 2^−ΔΔCT^ method as previously described [71]. Statistical analysis was performed in R and differences in gene expression were evaluated with Student’s t-test or one-way ANOVA (analysis of variance) followed by a Tukey post hoc test.

## Supplementary Information

**Additional file 1 **: **Table S1**. The origin of the wheat accessions used in this study and their population structure and principal component.

**Additional file 2 **: **Fig. S1.** Frequency distribution of the observed phenotypic data in 287 wheat genotypes. (a) survival; winter survival rate (0: 0-10% to 9: 90-100%), (b) DTH; days to heading, (c) DTM; days to maturity, (d) SL; spike length (cm), (e) SPL; spike length (cm), (f) AL; awn length (cm), (g) LW: liter weight (g), (h) TKW; thousand-kernel weight (g), (i) SPS: Number of seeds per spike. E1: Deokso in 2018-2019 season, E2: Deokso in 2019-2020 season, E3: Jeonju in 2018-2019 season, E4: Jinju in 2018-2019 season.

**Additional file 3 **: **Table S2**. Analysis of variance (ANOVA) of the nine agronomic traits in wheat genotypes (**p < 0.0001).

**Additional file 4 **: **Table S3**. The information of SNP markers used to analyze marker-trait associations.

**Additional file 5 **: **Fig. S2.** Q-Q plot and minor allele frequency (MAF) plot of SNPs associated with nine agronomic traits. In the Q-Q plot, X-axis and Y-axis represent cumulative *P*-values and observed *P*-values on a -log10 scale, respectively. In the MAF plot, each SNP is shown as circles and X-axis and Y-axis represent the MAF and the significance of marker-trait association, respectively. (a) winter survival rate, (b) days to heading, (c) days to maturity, (d) stem length, (e) spike length, (f) awn length, (g) liter weight, (h) thousand-kernel weight, (I) the number of seeds per spike.

**Additional file 6 **: **Fig. S3.***In silico* gene expression of the putative agronomic traits-related genes (a) under cold temperature and (b) in diverse plant tissues. DTH; days to heading, DTM; days to maturity, SL; stem length, SPL; spike length, AL; awn length, SPS; number of seeds per spike.

**Additional file 7 **: **Table S4**. The significant marker-trait associations (*P*-value < 0.001) for nine agronomic traits. he duplicated markers for each trait in different environments are marked in red.

**Additional file 8 **: **Table S5**. The information of real-time PCR primers used in this study.

**Additional file 9 **: **Fig. S4** Scatter plot of the linkage disequilibrium (LD) decay with the critical r^2^ value and the physical chromosome distance (Mbp) for the whole genome. The red dashed line shows the critical r^2^ value i.e., 0.34. The pink, green, blue, and red lines indicate LD decay of A, B, D, and all chromosomes, respectively. The LD was estimated as pairwise squared correlations of allele frequencies (r^2^) in sliding windows of 100 loci.

**Additional file 10 **: **Fig. S5.** Relative gene expression of winter survival rate-related candidate genes under cold treatment. Error bars indicate the standard error of the mean (*n* = 3). Asterisks indicate significant difference between the expression level of control and cold-treated plants (*p < 0.1, **p < 0.01).

**Additional file 11 **: **Fig. S6.** Relative gene expression of candidate genes associated with (a) days to heading, (b) days to maturity, (c) stem length, (d) spike length, (e) awn length, and (f) number of seeds per spike. Error bars indicate the standard error of the mean (*n* = 3). Different letters above each bar represent statistically significant differences (*P* < 0.05) after an analysis of variance (ANOVA) and a Tukey post hoc test.

## Data Availability

All data generated or analyzed during this study are included in this published article and its supplementary information files.

## Abbreviations

GWA: Genome-wide association
DTH: Days to heading
DTM: Days to maturity
SL: Stem length
SPL: Spike length
AL: Awn length
LW: Liter weight
TKW: Thousand kernel weight
SPS: The number of seeds per spike
BLUP: Best linear unbiased prediction
MLM: Mixed linear model
MTA: Marker-trait association
SSR: Simple sequence repeat
RAPD: Random amplified polymorphic DNA
RFLP: Restriction fragment length polymorphism
NGS: Next-generation sequencing
SNP: Single nucleotide polymorphism
CV: Coefficient of variation
ANOVA: Analysis of variance
MCMC: Markov chain Monte Carlo
PCA: Principal component analyses
REML: Residual maximum likelihood
IWGSC: International Wheat Genome Sequence Consortium
TPM: Transcripts Per Million
CDPK: Calcium-dependent protein kinase
MAPK: Mitogen-activated protein kinase
