# Supplementary material for: Identification of genetic loci associated with major agronomic traits of wheat (Triticum aestivum L.) based on genome-wide association analysis
Source: BMC Plant Biol. 2021 Sep 13;21:418. doi: 10.1186/s12870-021-03180-6 (PMC8436466; doi:10.1186/s12870-021-03180-6)
Supplement: Supplementary file 2 — Additional file 2 : Fig. S1. Frequency distribution of the observed phenotypic data in 287 wheat genotypes. (a) survival; winter survival rate (0: 0-10% to 9: 90-100%), (b) DTH; days to heading, (c) DTM; days to maturity, (d) SL; spike length (cm), (e) SPL; spike length (cm), (f) AL; awn length (cm), (g) LW: liter weight (g), (h) TKW; thousand-kernel weight (g), (i) SPS: Number of seeds per spike. E1: Deokso in 2018-2019 season, E2: Deokso in 2019-2020 season, E3: Jeonju in 2018-2019 season, E4: Jinju in 2018-2019 season. [file 12870_2021_3180_MOESM2_ESM.docx]

**Identification of Genetic Loci Associated with Major Agronomic Traits of Wheat (*Triticum aestivum* L.) Based on Genome-wide Association Analysis**

*BMC Plant Biology*

Woo Joo Jung^1^ , Yong Jin Lee^2^, Chon-Sik Kang^3^, Yong Weon Seo^1,2*^

^1^Department of Plant Biotechnology, Korea University, Seoul 02841, Korea

^2^Department of Biotechnology, Korea University, Seoul 02841, Korea

^3^National Institute of Crop Science, Rural Development Administration, Wanju 55365, Republic of Korea

*Corresponding author - Yong Weon Seo

E-mail: [seoag@korea.ac.kr](mailto:seoag@korea.ac.kr)


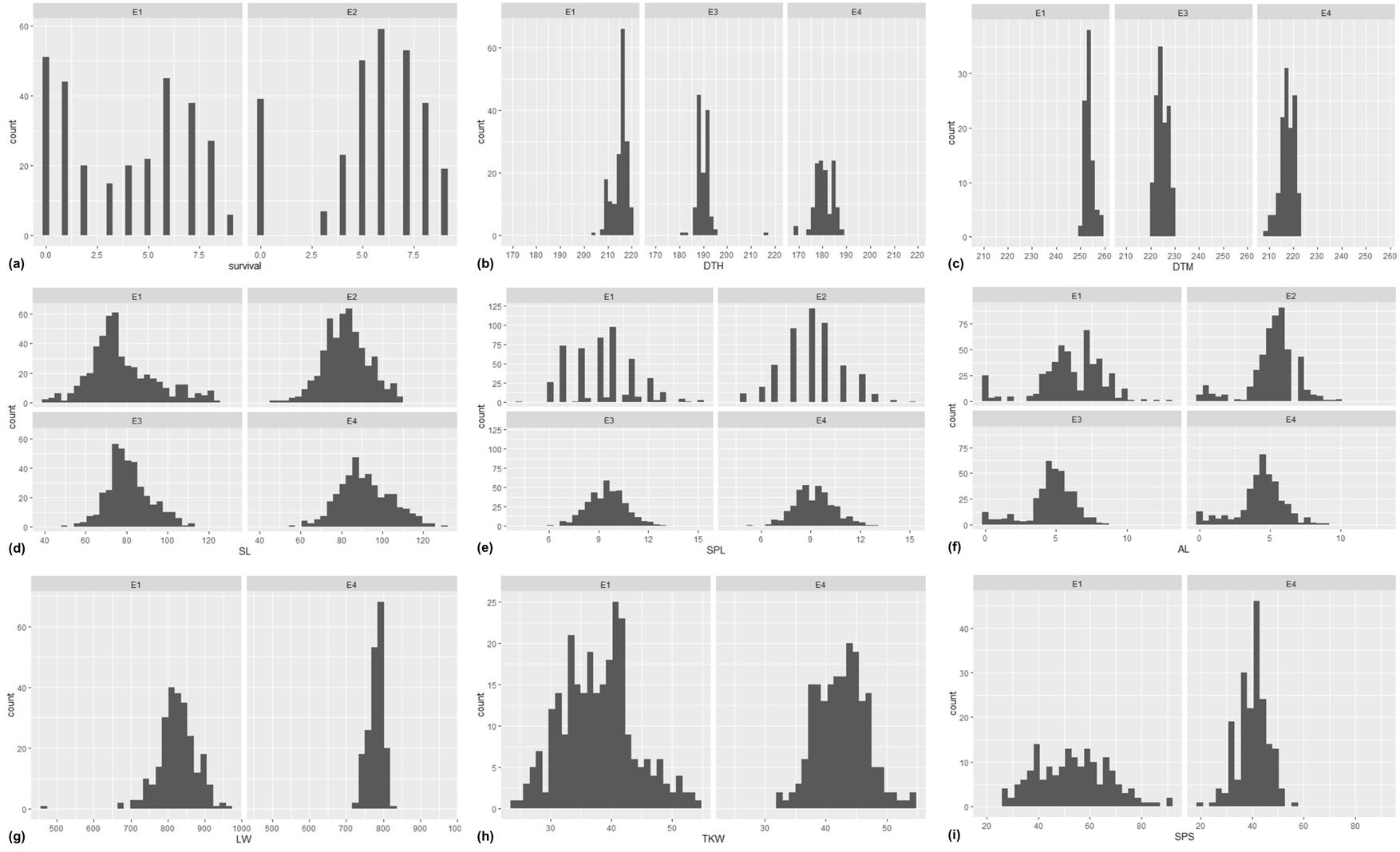


**Fig. S1** Frequency distribution of the observed phenotypic data in 287 wheat genotypes. (a) survival; winter survival rate (0: 0-10% to 9: 90-100%), (b) DTH; days to heading, (c) DTM; days to maturity, (d) SL; spike length (cm), (e) SPL; spike length (cm), (f) AL; awn length (cm), (g) LW: liter weight (g), (h) TKW; thousand-kernel weight (g), (i) SPS: Number of seeds per spike. E1: Deokso in 2018-2019 season, E2: Deokso in 2019-2020 season, E3: Jeonju in 2018-2019 season, E4: Jinju in 2018-2019 season
