# Supplementary material for: Identification of genetic loci associated with major agronomic traits of wheat (Triticum aestivum L.) based on genome-wide association analysis
Source: BMC Plant Biol. 2021 Sep 13;21:418. doi: 10.1186/s12870-021-03180-6 (PMC8436466; doi:10.1186/s12870-021-03180-6)
Supplement: Supplementary file 3 — Additional file 3 : Table S2. Analysis of variance (ANOVA) of the nine agronomic traits in wheat genotypes (**p < 0.0001). [file 12870_2021_3180_MOESM3_ESM.docx]

**Identification of Genetic Loci Associated with Major Agronomic Traits of Wheat (*Triticum aestivum* L.) Based on Genome-wide Association Analysis**

*BMC Plant Biology*

Woo Joo Jung^1^ , Yong Jin Lee^2^, Chon-Sik Kang^3^, Yong Weon Seo^1,2*^

^1^Department of Plant Biotechnology, Korea University, Seoul 02841, Korea

^2^Department of Biotechnology, Korea University, Seoul 02841, Korea

^3^National Institute of Crop Science, Rural Development Administration, Wanju 55365, Republic of Korea

*Corresponding author - Yong Weon Seo

E-mail: [seoag@korea.ac.kr](mailto:seoag@korea.ac.kr)

Table S2. Analysis of variance (ANOVA) of the nine agronomic traits in wheat genotypes (***p* < 0.0001)

|  |  | ***df*** | **Winter survival rate** | ***df*** | **Days to heading** | ***df*** | **Days to maturity** | ***df*** | **Stem length** | ***df*** | **Spike length** |
| --- | --- | --- | --- | --- | --- | --- | --- | --- | --- | --- | --- |
| **Type III Sum of Square** | **Genotype** | 286 | 5540.2 | 286 | 80813 | 188 | 22518 | 286 | 216386 | 286 | 2493.24 |
|  | **Environment** | 1 | 98.6 | 2 | 221774 | 2 | 199670 | 3 | 29956 | 3 | 73.94 |
|  | **Genotype* Environment** | 286 | 2342.2 | 346 | 3873 | 319 | 1475 | 571 | 54789 | 579 | 821.23 |
| **Mean Square** | **Genotype** | 286 | 19.304 | 286 | 282 | 188 | 120 | 286 | 756.96 | 286 | 8.7176 |
|  | **Environment** | 1 | 98.584 | 2 | 110887 | 2 | 99835 | 3 | 9985.4 | 3 | 24.6467 |
|  | **Genotype* Environment** | 286 | 8.161 | 346 | 11 | 319 | 5 | 571 | 96.0 | 579 | 1.4184 |
| **F Value** | **Genotype** | 286 | 15.0563**** | 286 | 284.26**** | 188 | 2.3955e+02**** | 286 | 29.3582**** | 286 | 9.1655**** |
|  | **Environment** | 1 | 76.8917**** | 2 | 111942.83**** | 2 | 1.9967e+05**** | 3 | 387.4621**** | 3 | 25.9129**** |
|  | **Genotype* Environment** | 286 | 6.3651**** | 346 | 11.30**** | 319 | 9.2502e+00**** | 571 | 3.7232**** | 579 | 1.4912**** |

Table S2. (Continued)

|  |  | ***df*** | **Awn length** | ***df*** | **Liter weight** | ***df*** | **Thousand kernel weight** | ***df*** | **Sees per spike** |
| --- | --- | --- | --- | --- | --- | --- | --- | --- | --- |
| **Type III Sum of Square** | **Genotype** | 286 | 3709.1 | 286 | 905632 | 286 | 23155.7 | 187 | 49291 |
|  | **Environment** | 3 | 743.5 | 1 | 470964 | 2 | 723.8 | 1 | 51753 |
|  | **Genotype* Environment** | 578 | 1850.0 | 150 | 492611 | 168 | 4877.0 | 168 | 24964 |
| **Mean Square** | **Genotype** | 286 | 12.969 | 286 | 3167 | 286 | 80.96 | 187 | 264 |
|  | **Environment** | 3 | 247.840 | 1 | 470964 | 2 | 361.90 | 1 | 51753 |
|  | **Genotype* Environment** | 578 | 3.201 | 150 | 3284 | 168 | 29.03 | 168 | 149 |
| **F Value** | **Genotype** | 286 | 29.3756**** | 286 | 63.331**** | 286 | 162.139**** | 187 | 21.186**** |
|  | **Environment** | 3 | 561.3730**** | 1 | 9419.270**** | 2 | 724.750**** | 1 | 4159.697**** |
|  | **Genotype* Environment** | 578 | 7.2499**** | 150 | 65.681**** | 168 | 58.135**** | 168 | 11.944**** |
