# Supplementary material for: Identification of genetic loci associated with major agronomic traits of wheat (Triticum aestivum L.) based on genome-wide association analysis
Source: BMC Plant Biol. 2021 Sep 13;21:418. doi: 10.1186/s12870-021-03180-6 (PMC8436466; doi:10.1186/s12870-021-03180-6)
Supplement: Supplementary file 5 — Additional file 5 : Fig. S2. Q-Q plot and minor allele frequency (MAF) plot of SNPs associated with nine agronomic traits. In the Q-Q plot, X-axis and Y-axis represent cumulative P-values and observed P-values on a -log10 scale, respectively. In the MAF plot, each SNP is shown as circles and X-axis and Y-axis represent the MAF and the significance of marker-trait association, respectively. (a) winter survival rate, (b) days to heading, (c) days to maturity, (d) stem length, (e) spike length, (f) awn length, (g) liter weight, (h) thousand-kernel weight, (I) the number of seeds per spike. [file 12870_2021_3180_MOESM5_ESM.docx]

**Identification of Genetic Loci Associated with Major Agronomic Traits of Wheat (*Triticum aestivum* L.) Based on Genome-wide Association Analysis**

*BMC Plant Biology*

Woo Joo Jung^1^ , Yong Jin Lee^2^, Chon-Sik Kang^3^, Yong Weon Seo^1,2*^

^1^Department of Plant Biotechnology, Korea University, Seoul 02841, Korea

^2^Department of Biotechnology, Korea University, Seoul 02841, Korea

^3^National Institute of Crop Science, Rural Development Administration, Wanju 55365, Republic of Korea

*Corresponding author - Yong Weon Seo

E-mail: [seoag@korea.ac.kr](mailto:seoag@korea.ac.kr)


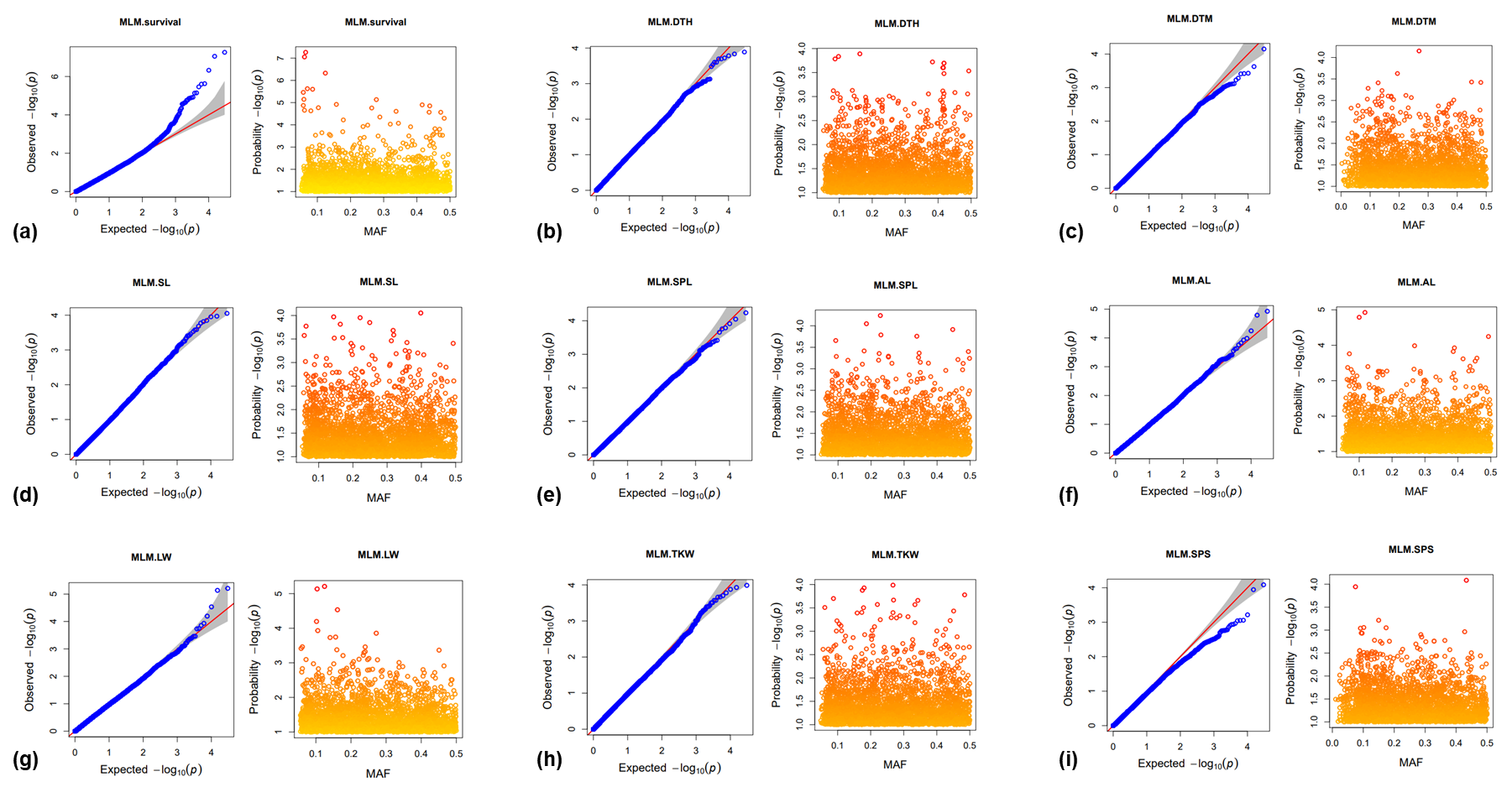


**Fig. S2** Q-Q plot and minor allele frequency (MAF) plot of SNPs associated with nine agronomic traits. In the Q-Q plot, X-axis and Y-axis represent cumulative *P*-values and observed *P*-values on a -log10 scale, respectively. In the MAF plot, each SNP is shown as circles and X-axis and Y-axis represent the MAF and the significance of marker-trait association, respectively. (a) winter survival rate, (b) days to heading, (c) days to maturity, (d) stem length, (e) spike length, (f) awn length, (g) liter weight, (h) thousand-kernel weight, (I) the number of seeds per spike
