# Supplementary material for: Identification of genetic loci associated with major agronomic traits of wheat (Triticum aestivum L.) based on genome-wide association analysis
Source: BMC Plant Biol. 2021 Sep 13;21:418. doi: 10.1186/s12870-021-03180-6 (PMC8436466; doi:10.1186/s12870-021-03180-6)
Supplement: Supplementary file 6 — Additional file 6 : Fig. S3.In silico gene expression of the putative agronomic traits-related genes (a) under cold temperature and (b) in diverse plant tissues. DTH; days to heading, DTM; days to maturity, SL; stem length, SPL; spike length, AL; awn length, SPS; number of seeds per spike. [file 12870_2021_3180_MOESM6_ESM.docx]

**Identification of Genetic Loci Associated with Major Agronomic Traits of Wheat (*Triticum aestivum* L.) Based on Genome-wide Association Analysis**

*BMC Plant Biology*

Woo Joo Jung^1^ , Yong Jin Lee^2^, Chon-Sik Kang^3^, Yong Weon Seo^1,2*^

^1^Department of Plant Biotechnology, Korea University, Seoul 02841, Korea

^2^Department of Biotechnology, Korea University, Seoul 02841, Korea

^3^National Institute of Crop Science, Rural Development Administration, Wanju 55365, Republic of Korea

*Corresponding author - Yong Weon Seo

E-mail: [seoag@korea.ac.kr](mailto:seoag@korea.ac.kr)


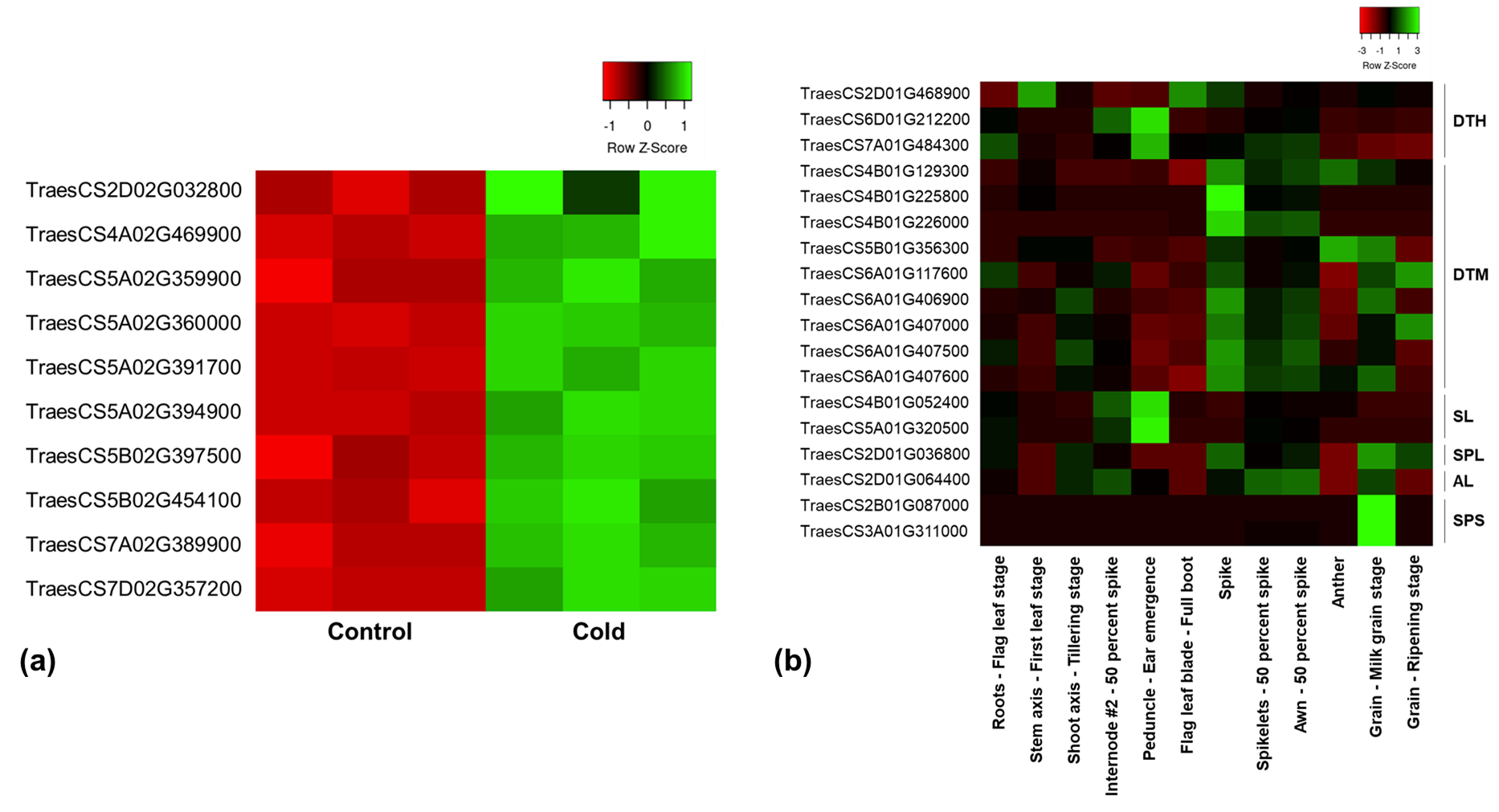


**Fig. S3** *In silico* gene expression of the putative agronomic traits-related genes (a) under cold temperature and (b) in diverse plant tissues. DTH; days to heading, DTM; days to maturity, SL; stem length, SPL; spike length, AL; awn length, SPS; number of seeds per spike
