# Supplementary material for: Identification of genetic loci associated with major agronomic traits of wheat (Triticum aestivum L.) based on genome-wide association analysis
Source: BMC Plant Biol. 2021 Sep 13;21:418. doi: 10.1186/s12870-021-03180-6 (PMC8436466; doi:10.1186/s12870-021-03180-6)
Supplement: Supplementary file 9 — Additional file 9 : Fig. S4 Scatter plot of the linkage disequilibrium (LD) decay with the critical r2 value and the physical chromosome distance (Mbp) for the whole genome. The red dashed line shows the critical r2 value i.e., 0.34. The pink, green, blue, and red lines indicate LD decay of A, B, D, and all chromosomes, respectively. The LD was estimated as pairwise squared correlations of allele frequencies (r2) in sliding windows of 100 loci. [file 12870_2021_3180_MOESM9_ESM.docx]

**Identification of Genetic Loci Associated with Major Agronomic Traits of Wheat (*Triticum aestivum* L.) Based on Genome-wide Association Analysis**

*BMC Plant Biology*

Woo Joo Jung^1^ , Yong Jin Lee^2^, Chon-Sik Kang^3^, Yong Weon Seo^1,2*^

^1^Department of Plant Biotechnology, Korea University, Seoul 02841, Korea

^2^Department of Biotechnology, Korea University, Seoul 02841, Korea

^3^National Institute of Crop Science, Rural Development Administration, Wanju 55365, Republic of Korea

*Corresponding author - Yong Weon Seo

E-mail: [seoag@korea.ac.kr](mailto:seoag@korea.ac.kr)


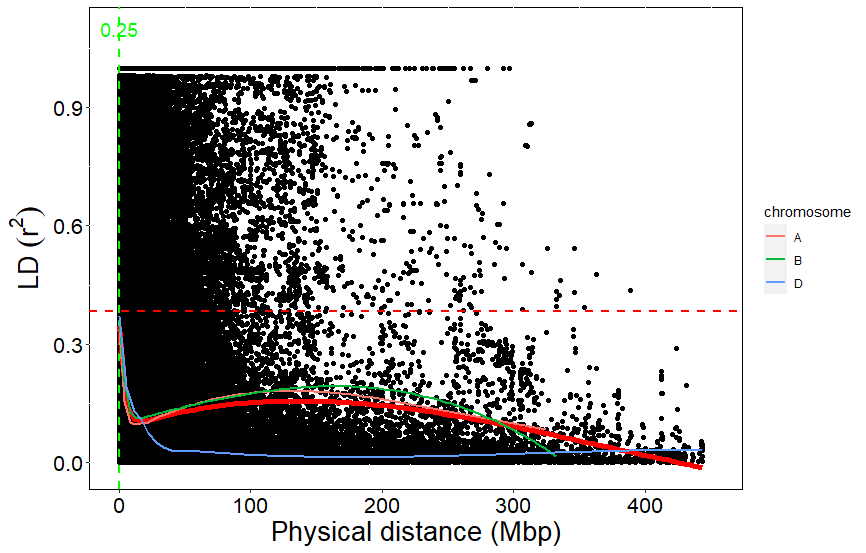


**Fig. S4** Scatter plot of the linkage disequilibrium (LD) decay with the critical r^2^ value and the physical chromosome distance (Mbp) for the whole genome. The red dashed line shows the critical r^2^ value i.e., 0.34. The pink, green, blue, and red lines indicate LD decay of A, B, D, and all chromosomes, respectively. The LD was estimated as pairwise squared correlations of allele frequencies (r^2^) in sliding windows of 100 loci.
