# Supplementary material for: Identification of genetic loci associated with major agronomic traits of wheat (Triticum aestivum L.) based on genome-wide association analysis
Source: BMC Plant Biol. 2021 Sep 13;21:418. doi: 10.1186/s12870-021-03180-6 (PMC8436466; doi:10.1186/s12870-021-03180-6)
Supplement: Supplementary file 10 — Additional file 10 : Fig. S5. Relative gene expression of winter survival rate-related candidate genes under cold treatment. Error bars indicate the standard error of the mean (n = 3). Asterisks indicate significant difference between the expression level of control and cold-treated plants (*p < 0.1, **p < 0.01). [file 12870_2021_3180_MOESM10_ESM.docx]

**Identification of Genetic Loci Associated with Major Agronomic Traits of Wheat (*Triticum aestivum* L.) Based on Genome-wide Association Analysis**

*BMC Plant Biology*

Woo Joo Jung^1^ , Yong Jin Lee^2^, Chon-Sik Kang^3^, Yong Weon Seo^1,2*^

^1^Department of Plant Biotechnology, Korea University, Seoul 02841, Korea

^2^Department of Biotechnology, Korea University, Seoul 02841, Korea

^3^National Institute of Crop Science, Rural Development Administration, Wanju 55365, Republic of Korea

*Corresponding author - Yong Weon Seo

E-mail: [seoag@korea.ac.kr](mailto:seoag@korea.ac.kr)


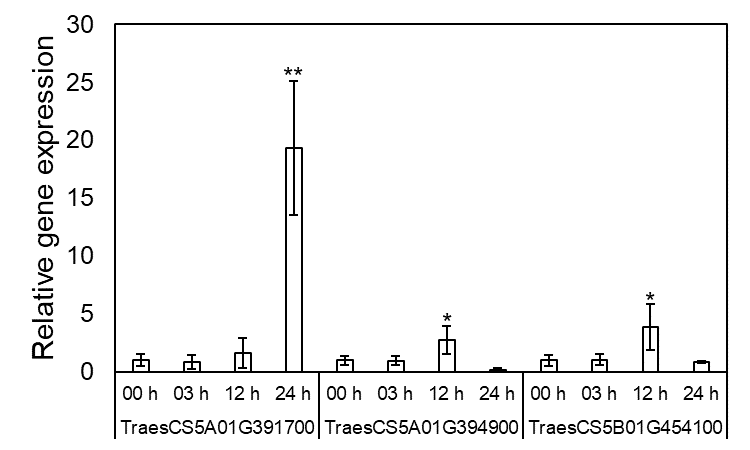


**Fig. S5** Relative gene expression of winter survival rate-related candidate genes under cold treatment. Error bars indicate the standard error of the mean (n = 3). Asterisks indicate significant difference between the expression level of control and cold-treated plants (**p* < 0.1, ***p* < 0.01).
