# Supplementary material for: Identification of genetic loci associated with major agronomic traits of wheat (Triticum aestivum L.) based on genome-wide association analysis
Source: BMC Plant Biol. 2021 Sep 13;21:418. doi: 10.1186/s12870-021-03180-6 (PMC8436466; doi:10.1186/s12870-021-03180-6)
Supplement: Supplementary file 11 — Additional file 11 : Fig. S6. Relative gene expression of candidate genes associated with (a) days to heading, (b) days to maturity, (c) stem length, (d) spike length, (e) awn length, and (f) number of seeds per spike. Error bars indicate the standard error of the mean (n = 3). Different letters above each bar represent statistically significant differences (P < 0.05) after an analysis of variance (ANOVA) and a Tukey post hoc test. [file 12870_2021_3180_MOESM11_ESM.docx]

**Identification of Genetic Loci Associated with Major Agronomic Traits of Wheat (*Triticum aestivum* L.) Based on Genome-wide Association Analysis**

*BMC Plant Biology*

Woo Joo Jung^1^ , Yong Jin Lee^2^, Chon-Sik Kang^3^, Yong Weon Seo^1,2*^

^1^Department of Plant Biotechnology, Korea University, Seoul 02841, Korea

^2^Department of Biotechnology, Korea University, Seoul 02841, Korea

^3^National Institute of Crop Science, Rural Development Administration, Wanju 55365, Republic of Korea

*Corresponding author - Yong Weon Seo

E-mail: [seoag@korea.ac.kr](mailto:seoag@korea.ac.kr)


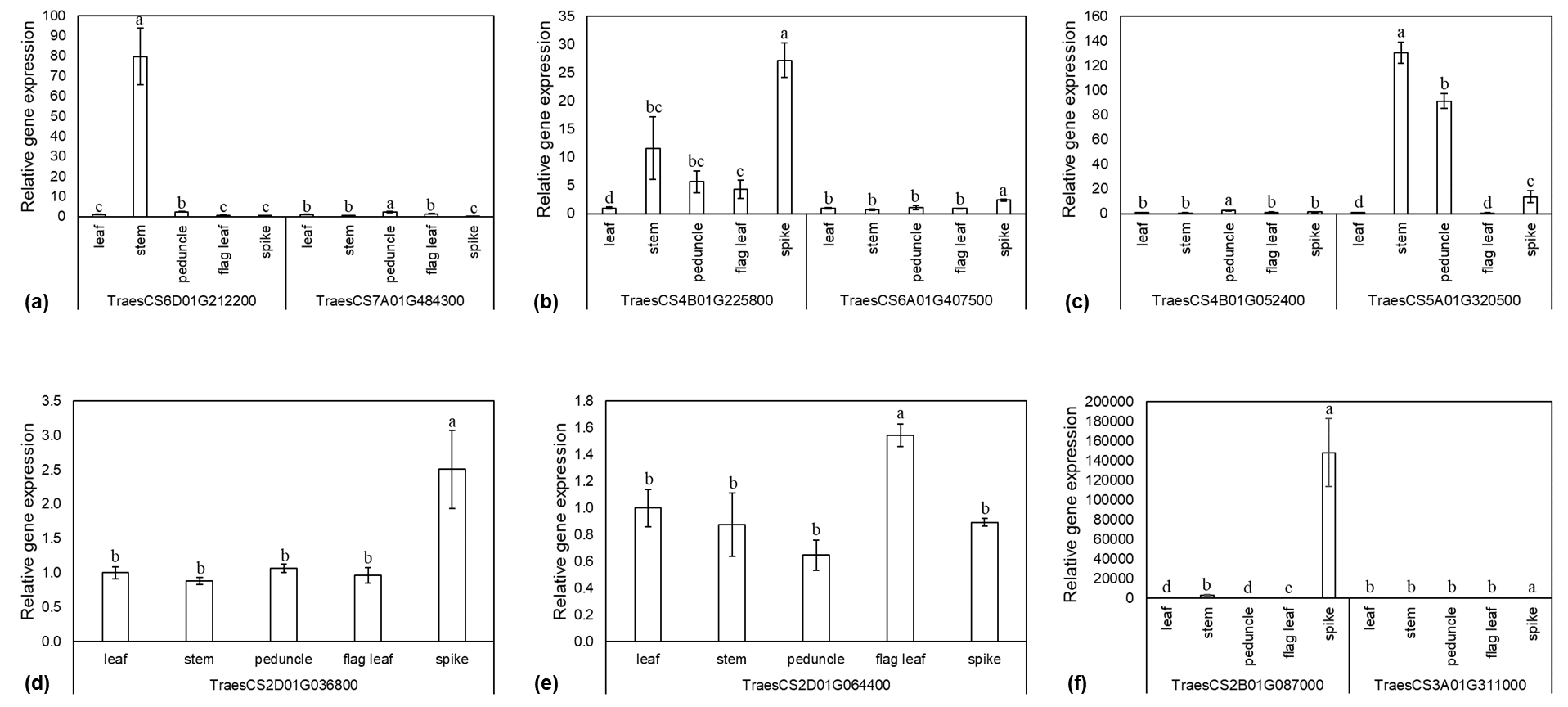


**Fig. S6** Relative gene expression of candidate genes associated with (a) days to heading, (b) days to maturity, (c) stem length, (d) spike length, (e) awn length, and (f) number of seeds per spike. Error bars indicate the standard error of the mean (n = 3). Different letters above each bar represent statistically significant differences (P < 0.05) after an analysis of variance (ANOVA) and a Tukey post hoc test.
